# Study on Prevalence of Trypanosomosis in Cattle of Sodo Zuriya District, Wolaita Zone, Southern Ethiopia

**DOI:** 10.1155/2021/4472480

**Published:** 2021-12-10

**Authors:** Nato Hundessa, Eshetu Esrael, Haben Fesseha, Mesfin Mathewos

**Affiliations:** School of Veterinary Medicine, Wolaita Sodo University, P.O. Box 138, Wolaita Sodo, Ethiopia

## Abstract

**Background:**

Trypanosomosis is a significant impediment to Ethiopia's livestock and farm production, adding adversely to the overall growth of agriculture in general and the nation's food self-reliance efforts in particular.

**Methods:**

Cross-sectional research was performed on the prevalence of trypanosomosis in cattle and the distinction of *Trypanosoma* species and potential factors in six selected provinces of the Sodo zuriya district in southern Ethiopia. Blood samples from the ear vein of 400 local and exotic cattle species were taken randomly. A hematocrit reader was used after centrifugation at 12,000 rpm for a five-minute assessment of anemic conditions of animals, and the anemia of the thin blood spraying and buffy coat procedure was tested to assess the incidence, detection, and packaging of *Trypanosoma* and the cell volume of each sampled animal.

**Results:**

Accordingly, twenty cattle were positive for trypanosomosis, with an overall proportion of 5.0%. From this overall prevalence, *Trypanosoma congolense* (3.3%) and *Trypanosoma vivax* (1.8%) were the two common *Trypanosoma* species in this study. The highest and the lowest prevalence of trypanosomes occurred in Guttuto Larena (35%) and Dalbo Wogane (0.00%), respectively. Age-wise prevalence revealed that young adults (45%) were the most affected, followed by old adults (35%) and adults (20%). Cattle with poor body condition scores (65%) were the most affected, followed by cattle with medium (30%) and good (5%) body condition scores, and this was found to be statistically significant (*p* ≤ 0.002). In addition, the variation in packed cell volume (PCV) between infected and noninfected cattle was significantly different (*p* ≤ 0.0001).

**Conclusion:**

Thus, the present study revealed the predominance of bovine trypanosomosis in the region and had a significant effect on body condition and anemia growth. The government and public should then collaborate in parasite-observed areas on the disease's management and prevention efforts in an environmentally sustainable way.

## 1. Introduction

Trypanosomosis is the primary hemoparasitic condition caused by unicellular protozoan parasites and reproduces in the bloodstream, lymphatic vessels, and tissue, together with cardiac muscles and the central nervous system [1]. It is one of the greatest barriers to animal production in Africa that is likely to increase the productivity of domestic livestock [2, 3].

Trypanosomosis in cattle and its vectors in vast areas in sub-Saharan Africa have devastating effects on livestock development and pose substantial threats to the survival of communities [1, 4]. Tsetse flies exist over ten million square kilometers in Africa, representing 38 countries in total [5, 6]. Recently, out of the total 147 million species of animals, approximately three hundred seventy-seven thousand species have been susceptible to tsetses in different countries. The European region produces 70 times more animal protein than the African region as a result of this disease [7, 8]. The gross losses in Africa are estimated at $500 billion annually [1, 9].

The main vector of trypanosomosis is tsetse flies, which are categorized under the genus *Glossina* species. In addition, *G. morsitans* is frequently identified in the savanna area, whereas *G. palpalis* favors rivers and lake areas and *G. fusca* is found in dense forest zones. These three species of *Glossina* convey trypanosomosis in various mammals [10, 11]. Moreover, biting flies can serve as mechanical vectors that involve the transfer of blood from one animal to another harboring infectious trypanosomes. Although biting flies are of huge importance in Africa, their role has not yet been established. The main mechanical vectors of *T. vivax* are Tabanus and other biting flies [10, 12].

More than one *Glossina* species (tsetse flies) is found in five regions of Ethiopia, namely, the Amhara area, Benishangul Gumuz, Gambella, Oromia, and Southern Nations, Nationalities, and Peoples' Regional State [13]. Accordingly, approximately 220,000 km^2^ of these regions are infested with tsetse fly species, namely, *Glossina pallidipes*, *G. morsitans*, *G. fuscipes*, *G. tachinoides*, and *G. longipennis* [14, 15].

Trypanosomosis in Ethiopia is one of the key concerns for animal growth and farming that lead to inclusive agricultural production in general and especially efforts on the nation's food self-sufficiency. Trypanosomosis due to tsetse flies commonly occurs in the west and southwest of the country and is more than 200,000 km^2^ favorable for farming activity. These areas have a huge potential for livestock, including cattle (14 million), shoat (nearly 14 million), equine (approximately 7 million), and camels (1.8 million). All these animal species can be infected by trypanosomosis at any moment [5, 11].

In Ethiopia, *Trypanosoma congolense*, *T. vivax*, and *T. brucei* are the most common trypanosomes in cattle, sheep, and goats, whereas *T. evansi* is the most common trypanosome in camels and *T. equiperdium* is the most common trypanosome in horses [5]. There are three essential elements for the increased risk of trypanosomosis: vector distribution, trypanosome virulence, and host response [3, 11, 16]. Ethiopia has the largest prevalence in western, southern, southwest, and northern regions following the broader Abbay, Omo, Ghibe, and Baro River Basins as a result of trypanosomosis and among the most significant diseases limiting animal production and agricultural growth [17, 18]. Trypanosome distribution is complex because of climate change, environmental disturbances, and human interference [19].

**Figure 1:**
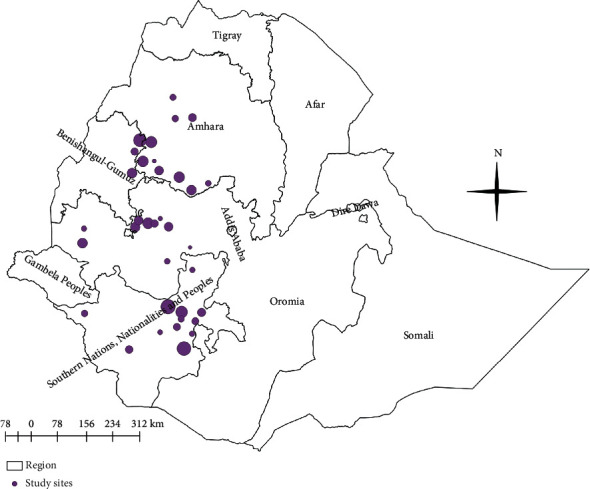
The spatial distribution of cattle trypanosomosis in different regions of Ethiopia [3].

The spatial distribution of bovine trypanosomosis found in various parts of Ethiopia is different. Most of the previous surveys were carried out in the western and southwestern parts of Ethiopia. Many published studies related to trypanosomosis have been found in various regional states, with a mean apparent prevalence of 8.17% in Amhara, 13.86% in Benishangul Gumuz, 6.34% in Oromia, and 7.91% in the Southern Nations, Nationalities, and Peoples' Regional State, whereas only a few published studies have been found in the Afar and Tigray regions [3] (Figure 1).

The most important estimation of the economic losses of trypanosomosis is based on mortality costs, lower weight gain in beef-type animals, lower milk returns, drawn production, and fertility effects [5, 11]. Furthermore, trypanosomosis is also responsible for the estimated loss of millions of dollars in animal revenue due to costs associated with attempts to manage, deter, and monitor vectors [1, 20]. In most southern regional parts of Ethiopia, trypanosomosis in cattle contributes to socioeconomic effects and decreases the development and productivity of animals via debilitation and increasing the mortality of infected animals [17, 18, 21]. Nevertheless, in the Sodo zuriya district in the Wolaita region, there is only a few recorded baseline evidence. The research was therefore performed in the Sodo zuriya district of the Wolaita zone to identify the prevalence and related risk factors for trypanosomosis in cattle.

## 2. Methods

### 2.1. Study Area

**Figure 2:**
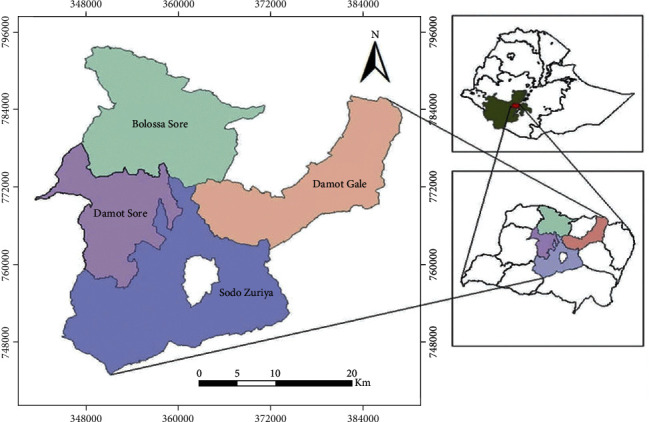
Map of the study area.

The present survey was carried out from October 2019 to April 2020 in the district of Sodo zuriya in the Wolaita Zone. The study site is located 390 km south of Addis Ababa and is found at 6°54′N latitude and 37°45′E longitude with an elevation between 1650 and 2980 meters above sea level. The district is bounded by the Damot Gale district to the north, Humbo district to the south, Damote Woyde district to the east, and Damote Sore district to the west; the annual rainfall and temperature of the area are 1000-1200 mm and 26-35°C, respectively. The site is classified under a midaltitude (“Woyina dega” in the local Amharic language) agroecological environment. The dry season lasts from September to February, and the rainy season remains from March to August. The livestock population of the region was estimated to be 1,097,710 cattle, 150,383 sheep, 185,250 goats, 60,055 equines, and 734,924 poultry [22] (Figure 2).

### 2.2. Study Animals

In this study, the study animals were local and exotic breeds of cattle of both sexes, various age groups, body conditions, and various hair coat colors that were managed under an extensive management scheme with communal herding. The study animals were classified as young, adult, or old based on dentition. Animals without erupted permanent incisor teeth were classified as young, while those with one or more pairs of erupted permanent incisor teeth were classified as adult and old [23, 24]. Based on the presence of the ribs and dorsal spines, the body condition score was determined to be good, medium, and poor [25]. The studied animals were split into five classes of white, red, black, and mixed coat colors to determine whether the coat color of the animal had any effect on the occurrence of the disease [26].

### 2.3. Study Design

Cross-sectional research was undertaken to assess the prevalence of trypanosomosis in cattle and estimate possible epidemiological risk factors.

### 2.4. Sample Size and Sampling Technique

The area was selected based on the available cattle population and ease of transportation and environmental conditions, and six kebeles (Peasant Association) from 34 kebeles (PAs) were identified and selected based on convenience. Animals from each locality were selected randomly to be included in the study. The sample size of this parasitological survey was calculated using the Thrusfield [27] formula.

(1) $$\begin{array}{c} n=\frac{1.96^{2}P_{\exp}\left(1-P_{\exp}\right)}{d^{2}}, \end{array}$$

where *n* is the required sample size, *P*_exp_ is the expected prevalence, and *d* is the desired absolute precision at 5%. Since there was no previous study undertaken in the specified study district, the expected prevalence was assumed to be 50%. Accordingly, the sample size computed was 384 cattle, but to increase the level of precision, the sample was increased to 400.

### 2.5. Parasitological and Hematological Investigations

#### 2.5.1. Physical Examination

The animal was examined by visualization of vital signs, such as the mucus membrane respiratory condition, heartbeat, pulse rate, body temperature, lymph nodes, and animal behavior (standing position and posture), and presence was examined before taking the sample.

#### 2.5.2. Blood Collection, Determination of Packed Cell Volume, and Thin Smear Preparation

After proper restraint, blood samples of 384 cattle were collected with heparinized capillary tubes from the marginal ear vein after disinfection with 70% alcohol and sealed with wax at one end for hematological analysis. Meanwhile, data of each cattle, such as age, sex, body condition score, and skin coat color, were recorded in the designed format. All parasitological diagnostic tests and procedures were conducted as described by Paris et al. [28].

The microhematocrit capillary tubes (up to 3/4th) were filled with blood with an outermost sealed end. The blood specimens were centrifuged at 12,000 rpm for 5 min using a hematocrit centrifuge. To assess the degree of anemia, the packed cell volume was measured using a hematocrit reader. The capillary tubes were then removed via a diamond-tipped pen 1 mm below the buffy coat to include the uppermost layers of the red blood cells and 3 mm above to include the plasma. The content was expressed on a clean microscopic slide, mixed well, and covered with a 22 × 22 mm coverslip [29]. After centrifugation, the capillary tubes were placed in a hematocrit reader to determine packed cell volume (PCV) expressed as a percentage of the total volume of blood, taking PCV values ≥ 24 to 46% as normal for zebu cattle [30, 31].

During wet smearing, a 40x microscope objective lens was used to identify motile trypanosomes, and the different types of species were confirmed through Giemsa staining at 100x magnification on the basis of their morphology. For positive cases, in Giemsa-stained blood smears, the morphology of the species can be distinguished by their size, shape, location, size of the kinetoplast, position of nucleus, and attachment and length of the flagellum. *T. vivax* was represented by monomorphic forms in which a free flagellum was always present. In addition, the posterior end of the body is typically rounded, and the kinetoplast is large and terminal with a nonprominent undulating membrane. *T. congolense* was represented by small forms in which free flagellum was absent. Additionally, the kinetoplast is marginal and of medium size with a nonprominent undulating membrane. *T. brucei* is represented by stout monomorphic forms with a short free flagellum, and the kinetoplast is subterminal and small in size with a prominent undulating membrane [28, 32].

### 2.6. Data Analysis

The data were entered into Microsoft Excel, and the prevalence of major trypanosomosis in cattle of various age categories and both sexes was analyzed via STATA software version 13 (Stata Corporation, College Station, USA). The relationship of potential risk factors and trypanosomosis was tested with the chi-square test. Differences between variables at 0.05 or less were checked for significance in all situations. Descriptive statistics were used for the interpretation of the results, such as frequency and proportion.

## 3. Results

### 3.1. Parasitological Findings

In the present study, out of the 400 examined cattle, 20 (5.0%) cattle were positive for trypanosomosis. *Trypanosoma congolense* and *Trypanosoma vivax* were the main species identified with 3.3% and 1.8% prevalence, respectively (Table 1).

### 3.2. Prevalence across the Selected Host-Related Risk Factors

Table 2 shows the prevalence of *Trypanosoma* infection among the breeds of cattle. Of the twenty positive animals, 1 (5%) exotic breed harbored *Trypanosoma* species, followed by 19 (95%) local breeds. No significant variation (*p* = 0.912) was observed between breeds and trypanosome infection. On the other hand, the highest prevalence of trypanosome infection was recorded in Guttuto Larena (35.00%), followed by Ansome Gembela (20%), Offa Gandaba (10%), Tome Gerera (15%), and Waciga Busha (20%). However, there was no significant difference (*p* = 0.117) in the prevalence among the study villages.

Age-wise prevalence revealed that old cattle (6.19%) were the most affected, followed by young cattle (5.73%) and adults (3.08%). However, males (5.79%) were the most affected compared to females (4.29%), and there was no significant difference (*p* = 0.49) between the sexes. Among infected cattle, 3.70% were mixed, 2.53% were white, 5.81% were red, and 5.74% were black. Depending on their body condition, cattle with poor body condition (10.66%) were the most infected, followed by medium (3.09%) and good (1.19%) cattle. This was statistically significant (*p* = 0.002) (Table 2).

### 3.3. Effect of Trypanosomosis on Packed Cell Volume (PCV)

The investigation of PCV results in the study cattle revealed that the mean PCV value for the parasitemic cattle was 21.47% (95%CI = 21.03 − 21.90%), which is lower than that of the mean PCV value for the aparasitemic animals at 24.2% (95%CI = 27.96 − 28.50). There was a statistically significant difference in the mean PCV value between the infected and noninfected animals (*t* = 27.13, *p* ≤ 0.001) (Table 3).

## 4. Discussion

The current investigation covers an overall of 384 randomly selected cattle, 20 (5%) of which are positive for trypanosome infection. *Trypanosoma congolense* (3.3%) and *Trypanosoma vivax* (1.8%) were the main species identified during hematological examination. Of these, 6.25% in Ansome Gambela, 0.00% in Dalbo Wogane, 10.94% in Guttuto Larena, 4.69% in Tome Gerera, 5.0% in Waciga Busha, and 3.13% in Offa Gandaba were recorded.

According to the sampled villages of the district, the highest prevalence was observed in Guttuto Larena (10.94%) village, and the lowest prevalence was observed in Dalbo Wogane (0.0%). Nevertheless, the current survey was higher than the earlier reports of Abebayehu et al. [33], Ayana et al. [34], and Lelisa et al. [35] who reported a prevalence of 2.66%, 2.10%, and 4.25% from Western Tigray, Arbaminch area, Northwest Ethiopian and Illuababora Zone, Southwestern Ethiopia, respectively. The discrepancies between reports may be focused on the differences in the method of management, the season for the research time, drug resistance growth, and the increasing vector density challenge due to the animal's lack of knowledge about the diseases in the field of study.

The prevalence of bovine trypanosomosis in the study district was 5%. This result is comparable with the previous prevalence of Teka et al. [26] who reported 4.43% in Arbaminch and Fayisa et al. [36] who reported 4.86% in the Didessa District. However, the present study was lower than that of Bishaw et al. [37] reported 7.8% in the Wemberma area of West Gojjam Zone, Ethiopia; Eshetu and Begejo [38] reported 8.3% in Mareka Woreda of Dawuro Zone, southern Ethiopia; and Kebede and Animut [39] reported 9.63% in Awi Zone, northwest Ethiopia. Begna et al. [40] reported 14.2% in selected villages of Humbo district, southern Ethiopia; Shimelis et al. [41] reported 19.01% in Ghibe Valley; Zeryehun and Abraham [42] who reported 27.5% in selected areas of Arba Minch, southern Ethiopia; Mulaw et al. [43] reported 28.1% in the tsetse-infested area of Assosa area of Benishangul Gumuz Regional State; Takele [44] reported 32% in Gamo Gofa; Amenu [45] reported 42.6% in Arbaminch Zuriya and Boreda Abaya; Amare [46] reported 40.5% in Damote Woyde woreda of Wolaita; Haile [47] reported 35.5% in North Omo; and Ademe and Abebe [48] reported 37% in Kindo Koyisha.

The reason for the reduction of the *Trypanosoma* was the presence of the Southern Valley Tsetse and Trypanosomosis Eradication Project (STEP), such as a chemical application on the back of the animals, ground spraying, insecticide-impregnated targets, and aerial spray that significantly reduces the occurrence of the disease in the survey site. The use of trypanocidal medications as a prophylactic agent was another explanation for the reduced incidence of the disease.

Two species of trypanosomes, primarily *Trypanosoma congolense* and *Trypanosoma vivax*, were described in this study. The most dominant trypanosome species in the present study was *Trypanosoma congolense*, which was 3.3%, and the less prevalent trypanosome species was *Trypanosoma vivax*, which was 1.8%. This was against the report of Sinshaw et al. [49] with a frequency of trypanosomosis due to *T. vivax* ranging from 4% to 9.6% in the three highland districts bordering Lake Tana. The current research was much lower than the previous finding of Ayele et al. [50] who reported 93% *T. congolense* and 5.3% *T. vivax* in the Daramallo area of southwestern Ethiopia, Efrem et al. [51] who reported 75% *T. congolense* and 25% *T. vivax* in the Lalo Kile District of the Kelem Wollega zone, and Desta [52] who reported 81.42% *T. congolense* and 12.85% *T. vivax* in the upper Didessa Valley of western Ethiopia. Moreover, all the above studies revealed that *T. congolense* was more prevalent than *T. vivax*, which was in agreement with the current finding.

In contrast to the present study, the findings of Kidanemariam and his colleagues in the Kindo Koyesha district [53] showed that *T. vivax* (71%) was the most prevalent species than *T. congolense* (28.4%). The high proportion of *T. congolense* in the present study might be due to its high number of biological vectors in the area compared to *T. vivax*. The lower prevalence of *T. vivax* might be due to the low distribution of mechanical vectors, such as Tabanus and *Musca domestica*.

In the present study, males (5.79%) were the most affected compared to females (4.29%), and there was no significant difference (*p* > 0.05) between the sexes. This finding was in agreement with Muturi [54] who reported that males have a higher prevalence of trypanosomosis than females. This occurred due to grazing conditions for male animals rather than females related to their physiological situations, and this study disagreed with previous results of Girma et al. [55] who obtained no significant difference in susceptibility between both males and females.

In this research, the occurrence of trypanosomosis in cattle was investigated in three different body conditions, including poor, good, and medium body-sized animals, and the maximum frequency was recorded in poor body condition (10.66%), followed by medium (3.09%) and good body conditions (1.19%). This study was highly in agreement with Eshetu et al. [56] in which they reported 23.3% in poor body conditioned, 5.5% in medium body conditioned, and 4.1% in good body conditioned animals, and another report made by Chanie et al. [57] reported 55.7% prevalence in poor and 6.7% prevalence in medium body condition score. Thus, cattle with poor body conditions have a high susceptibility to the disease because of immune suppression due to blood parasites [1].

In the present study, different skin or hair coat colors (white, red, black, and mixed) of the animals were considered a potential risk factor for the prevalence of trypanosomosis. Accordingly, a higher infection rate was observed in cattle with red skin color (5.81%), followed by black color (5.74%), mixed (3.70%), and white color (2.53%). This research was not in agreement with the report of Teka et al. [26] who stated a higher infection rate in varied hair coat color (7.25%), followed by red (4.88%), black (3.57%), white (1.56%), and gray (0.0%) skin color. Additionally, Eshetu et al. [56] reported that a higher infection rate was observed in black (25.6%), mixed (9.4%), red (9%), gray (2.9%), and white (2.6%) skin colors of animals. The study by Fetehanegest et al. [58] also revealed that the prevalence rate was higher in black (12.4%), red (5.17%), and white (3.23%) skin colors of animals. The possible reason for this finding is the high number of red cattle in the study area and the low nutritional factors and management system. On the other hand, naturally, tsetse flies are attracted to black skin color rather than other skin colors.

Age was assumed to be one of the determinant factors in the current study; accordingly, a higher rate of infection was recorded in old cattle (6.19%), followed by younger cattle (5.73%) and adult cattle (3.08%). This was comparable with the previous report of Begna et al. [40] in selected villages of Humbo District, southern Ethiopia; Dawud and Molalegne [59] in Benishangul Gumuz Regional State, western Ethiopia; and Molalegne et al. [60] in Jabi Tehenan district, northwestern Ethiopia, where higher prevalence was reported in adult and old animals. This may be associated with aged animals being used for farming, irrigation, and harvesting crops. Rowland et al. [61], in Ghibe Valley, described that suckling calves do not go out with their dams but sit home until they are weaned away. In addition, young animals are often shielded by maternal antibodies to a certain degree [62]. This may be why trypanosomosis was found in calves less prevalently. In adult species, *Trypanosoma congolense* is always higher than in younger animals [63].

The present study showed that the mean PCV value for parasitemic cattle was 21.47% (PCV ≤ 24%), which was lower than the mean PCV value for aparasitemic cattle at 24.2% (PCV > 24). The present result was comparable to the findings of Begna et al. [40] in Humbo District, southern Ethiopia; Molalegne et al. [60] in Jabi Tehenan district, northwestern Ethiopia; Dawud and Molalegne [59] in Mao-Komo, Benishangul Gumuz, western Ethiopia; Haile [47] in North Oromo; and Cherenet et al. [64] in Amhara Region, northwestern Ethiopia.

Considering the PCV value of 24-46% as normal for zebu cattle [30], 70% of the parasitemic and 40% of the aparasitemic animals have registered PCV values less than 24%. The resulting low PCV value may not solely be due to trypanosomosis; however, the difference in mean PCV between parasitemic and aparasitemic animals indicates that trypanosomosis reduces the PCV values in infected animals. These might be exacerbated by other diseases that are considered to reduce the PCV values in infected animals in the study area, such as helminthosis, tick-borne diseases, and nutritional imbalances. This indicates that even though trypanosomosis is indicative of anemia, other causes may also induce decreased PCV, but trypanosome infection could certainly retain their PCV within a normal range of time. Therefore, the diagnosis of trypanosomosis on the basis of PCV is not valid.

## 5. Conclusion

The present survey revealed that bovine trypanosomosis is a disease of economic significance that affects both the health and efficiency of cattle in Sodo Zuriya. The key trypanosome species in the field under study were *T. congolense* (3.3%), followed by *T. vivax* (1.8%). The prevalence of bovine trypanosomosis was affected by multiple possible risk factors. Thus, the prevalence of trypanosomosis was higher in males, poor body condition, and red-coated cattle. The mean PCV value for the parasitemic cattle was 21.47% (PCV ≤ 24%), which is less than that of the mean PCV value for the aparasitemic animals at 24.2% (PCV > 24). In conclusion, it is necessary to raise awareness of the effects of the disease and abuse of veterinary drugs among livestock owners and to include them in the diagnosis, reporting, and management of economically significant diseases. To regulate the supply of drugs to practitioners, the government can enact drug law policies. More studies should be conducted during various seasons of the year on the risk factors, drug resistance, and disease epidemiology.

## Figures and Tables

**Table 1:** Proportion of *Trypanosoma* species in the study sites.

Trypanosoma species	No. positive samples	Frequency	Percent (%)
*Trypanosoma congolense*	20	13	3.3
*Trypanosoma vivax*		7	1.8

**Table 2:** Prevalence of *Trypanosoma* infection among the potential risk factors.

Variables	Category	No. positive animals	Proportion (%)	Chi-square (*X*^2^)	*p* value
Breed	Local	19	4.97	0.012	0.91
	Exotic	1	5.56		

Age	Old	7	6.19	1.53	0.47
	Adult	4	3.08		
	Young	9	5.73		

Sex	Male	11	5.79	0.48	0.49
	Female	9	4.29		

Color	Black	7	5.74	1.49	0.69
	Red	10	5.81		
	White	2	2.53		
	Mixed color	1	3.70		

Body condition score	Good	1	1.19	12.27	0.002
	Medium	6	3.09		
	Poor	13	10.66		

Study sites	Ansome Gembela	4	6.25	8.82	0.117
	Dalbo Wogane	0	0.0		
	Guttuto Larena	7	10.94		
	Tome Gerera	3	4.69		
	Waciga Busha	4	5.00		
	Offa Gandaba	2	3.13		

**Table 3:** Mean PCV of trypanosome-infected and uninfected cattle in the study areas.

Status	No. examined	Anemic	Mean PCV ± SD	*t* value	*p* value
Parasitemic animals	137	19	21.47 ± 2.58	27.13	0.001
Aparasitemic animals	263	1	28.234 ± 2.25		

## Data Availability

The datasets used and analyzed during the current study are available from the corresponding author on reasonable request.

## Abbreviations

PCV: Packed cell volume
rpm: Revolutions per minute
STEP: Southern Valley Tsetse and Trypanosomosis Eradication Project.
